# Outcomes of patients with HIV and COVID-19 co-infection: a systematic review and meta-analysis

**DOI:** 10.1186/s12981-021-00427-y

**Published:** 2022-01-14

**Authors:** Celestin Danwang, Jean Jacques Noubiap, Annie Robert, Jean Cyr Yombi

**Affiliations:** 1 Epidemiology and Biostatistics Unit, Institut de Recherche Expérimentale et Clinique, Université Catholique de Louvain, Brussels, Belgium; 2 Centre for Heart Rhythm Disorders, University of Adelaide and Royal Adelaide Hospital, Adelaide, Australia; 3 Department of Internal Medicine and Infectious Diseases, HIV/AIDS Reference Center, Cliniques Universitaires Saint-Luc, Avenue Hippocrate 10, 1200 Brussels, Belgium

**Keywords:** Outcomes, HIV, Systematic review, Meta-analysis

## Abstract

**Background:**

Data on the association of human immunodeficiency virus (HIV) infection with adverse outcomes in patients with COVID-19 are conflicting. This systematic review and meta-analysis aimed to summarize the available information on the risk of hospitalization, severe disease, and death attributable to HIV in patients with COVID-19.

**Methods:**

PubMed, EMBASE, Web of Science, and SCOPUS were searched through October 25, 2021, to identify relevant studies, without language restriction. A random-effects model was used to pool estimates.

**Results:**

We included 44 studies reporting information from 38,971,065 patients with COVID-19. The pooled prevalence of HIV among COVID-19 patients was 26.9 ‰ (95% CI 22.7–31.3) and was significantly higher in studies conducted in Africa compared to those conducted elsewhere (118.5‰ [95% CI 84.8–156.9, 11 studies] vs 10.9‰ [95% CI 8.8–13.2, 27 studies]). In pooled analyses of unadjusted odds ratio, HIV-positive individuals were more likely to be admitted to hospital (OR: 1.49; 95% CI 1.01–2.21, 6 studies) compared to HIV-negative individuals. In the adjusted (for age and sex) analyses, HIV was associated with an increased risk of death (hazard ratio: 1.76, 95% CI 1.31–2.35, 2 studies). However, HIV was not associated with the severity of the disease (OR: 1.28; 95% CI 0.77–2.13, 13 studies), or death (OR: 0.81; 95% CI 0.47; 1.41, 23 studies) in patients with COVID-19 in the meta-analysis of unadjusted odds ratio.

**Conclusion:**

Our findings suggest that patients with HIV have an increased risk of hospital admission for COVID-19. HIV seems to be independently associated with increased risk of mortality in COVID-19 patient in adjusted analysis. However, this evidence was derived from only two studies.

**Supplementary Information:**

The online version contains supplementary material available at 10.1186/s12981-021-00427-y.

## Introduction

The coronavirus 2019 (COVID-19) pandemic is imposing to the world a huge health, societal and economic burden [1–3]. Despite all the efforts that have been made to reduce the spread of the virus and limit its lethality, the death rate from COVID-19 remains high. Indeed, as of 3 November 2021, approximately 246,951,274 cases of COVID-19 have been diagnosed worldwide and 5,004,855 associated deaths have been recorded [4]. Results from vaccination campaigns are promising, with a marked reduction in new infections regardless of the variant in vaccinated individuals compared to unvaccinated or partially vaccinated people [5–8].

Although all ages and profiles are likely to be affected by COVID-19, studies suggest that patients with co-morbidities are particularly at risk of adverse outcomes compared to those without [9–11]. For instance, patients with hypertension, obesity and diabetes are more likely to die, be admitted to intensive care units and have severe forms of the infection [9–11]. For some other diseases such HIV, the information on their association with adverse outcomes in patients with COVID-19 are conflicting [12–17]. However, some regions of the world like sub-Saharan Africa are at risk of having a burden of COVID-19 drive by the proportion of HIV patients. Sub-Saharan Africa for example bored the highest burden of HIV and patients not receiving antiretroviral therapy (ART) [18–21]. Among the 38 millions of patients living with HIV globally, 26 million are in this part of the continent, with a relatively high proportion not receiving ART compare to other region of the world [18, 19].

HIV causes immunodepression by depleting CD4 cells, thus reducing the capacity of the organism to defend against bacterial, fungal, parasitic, and viral infections such as COVID-19 [20, 22]. This vulnerability to infection is greater when the immunodepression is severe and the patient is not on ART making the patient at risk of opportunistic infections [23, 24]. The presence of 38 million people worldwide with HIV during this period of COVID-19, could therefore be challenging for health systems worldwide as more aggressive preventive and therapeutic measures might be needed for this population. It is therefore necessary for programmatic purposes, optimal allocation of public health interventions and prioritisation of care in a context of scarce resources due to the pandemic [25], to know whether, given the state of the art, people living with HIV are proportionally more affected than people without the disease, and whether they are at greater risk of pejorative outcome when affected by COVID-19.

Hence, this study aimed to summarize the available information on the risk of hospitalization, severe disease, and death attributable to HIV in patients with COVID-19 and to determine the proportion of patients co-infected with HIV among patients with COVID-19.

## Methods

This review is reported in accordance with the PRISMA guidelines and is registered with PROSPERO CRD42021255993.

### Search strategy and eligibility criteria

PubMed, EMBASE, Web Sciences, and SCOPUS were searched from 1 December 2019 to 25 October 2021 without language restriction for studies reporting the outcome of COVID-19 according to HIV status. Only studies involving patients with confirmed COVID-19 infection (polymerase chain reaction or rapid diagnostic test) were included. HIV positivity/negativity was defined as per reported by each study. Both study reporting on patients with a known HIV status prior to the COVID-19 pandemic, and those in which the diagnostic of HIV was made in patients with COVID-19 during hospitalisation were considered. Severe COVID-19 was defined as the presence of blood oxygen saturation ≤ 93%; multiple organ dysfunction; respiratory failure; septic shock; dyspnoea; respiratory rate greater than 30/min, PaO2/FiO2 ratio < 300, and/or lung infiltrates > 50% of the lung field within 24–48 h [26]. For duplicates or studies published in more than one report, or conducted on the same database, the one reporting the largest sample size was considered.

The detailed search strategy is presented in the Appendix (Additional file 1: Tables S1–S3). We included all studies with at least 20 participants in each group (with and without HIV) and reporting sufficient information to determine the number of hospital admissions, severe cases, or deaths in each group.

After removing duplicates, two investigators (CD, JJN) assessed the eligibility of the retrieved articles, first based on the title and abstract, then on full text. Disagreements between the two investigators were resolved by discussion and consensus.

### Data extraction and quality assessment

In each study, we extracted the name of the first author, the year of publication, the country, the characteristics of the study population (proportion of men, age distribution), the total number of patients with and without HIV in the study, the number of patients with each outcome between those with and without HIV. For all studies reporting prevalence data, the Joanna Briggs Institute (JBI) critical appraisal tool for prevalence studies was used to assess the risk of bias [27], with the following ranges 0–3, 4–6 and 7–9 indicating high, moderate and low risk of bias, respectively. For the remaining studies, the JBI tool corresponding to the study design was used [28].

### Statistical analysis

To obtain the overall proportion of HIV patients among COVID-19 patients, a DerSimonian-Laird random-effects model for meta-analysis within the "meta" package of R was performed. Then, to estimate the overall risk of hospital admission, severe COVID-19, and the risk of death among HIV and COVID-19 co-infected patients, a random-effects model was run. A subgroup analysis was performed according to country location (USA vs. Non-USA, and Africa vs. Non-Africa).

In addition, adjusted odds ratios (OR) or hazard ratio (HR) when available (with their standard error) were pooled to obtain an adjusted estimate for each outcome where there were at least two studies. We have assessed the association between mortality and death with the two ways of measuring methods of associations, the odds ratio and the hazard ratio. Cochran and I^2^ statistics were used to assess and estimate the degree of heterogeneity in the meta-analysis [29, 30]. I^2^ ranging from 0 to 40%, 40–75%, 75–100% was considered as indicative of low, moderate, and substantial heterogeneity respectively. Visual inspection of the funnel plot and Egger's test were used to assess publication bias. A sensitivity analysis was performed to detect influential studies. A p-value of ≤ 0.05 was considered statistically significant. All analyses were performed with R software, version 4.0.2.

## Results

### Study selection and characteristics

We found 8537 studies from literature searches, and finally included 44 studies reporting information from 38,971,065 patients with COVID-19 in the meta-analysis (Additional file 1: Fig. S1). Thirteen (41.9%) studies were conducted in the USA. Twenty-eight studies were cross-sectional studies, eight were cross-sectional analyses of a cohort study, three were case series and five were case controls. Twenty-eight of the 44 studies were multicentre. All studies included in the systematic review and meta-analysis were in English. The sex ratio, and age distribution of patients according to HIV status was greatly variable according to study as summarized in Table 1.

**Table 1:** Characteristics of studies included in the meta-analysis

Author	Year of publication	Country	Period of inclusion	State/city/region	Number of Centre	Registry	Setting	Mean/Median age (yrs.)	Min. age	Max. age	%Males	Nber of patients with COVID-19	Nber of patients with COVID-19 and HIV	RoB
Bakamutumaho	2021	Uganda	March to December 2020	Entebbe	Single-Site study	HR	HB	35 (IQR:27–47)	NR	NR	83	270	27	Low
Bhaskaran	2021	UK	Feb,1,2020	National	Multi-Site study	Yes	HB	HIV positive: 48 (40–55), HIV negative:49 (34–64)	18	NR	HIV positive: 64.7, HIV negative: 49.9	17,282,905	27,480	Low
Blanco	2020	Spain	March 9, 2020	Barcelona	Single-Site study	HR	HB	HIV positive: 37.8	HIV positive: 29	HIV positive: 49	60	543	5	Low
Boulle	2020	South Africa	1 March to 9 June 2020	Western Cape	Multi-Site study	HR	HB	NR	20	NR	HIV positive: 22.0, HIV negative 33.7	22,308	3978	Low
Braunstein	2020	USA	June 2, 2020	New York	Multi-Site study	Yes	PB	NR	0	NR	HIV positive:71.4, HIV negative 51.1	204,442	2410	Low
Byrd	2020	USA	30 March and 20 May 2020	Rhode Island	Single-Site study	Yes	PB	NR	30	71	74.1	150	27	Low
Cabello	2021	Spain	February 1 until May 20, 2020	Madrid	Multi-Site study	HR	HB	46 (IQR: 37–56)	NR	NR	88.9	7061	31	Low
Calza	2020	Italy	March 1, 2020, and April 30, 2020	Bologna	Single-Site study	HR	HB	53.8 (IQR: 42.5–64.7)	NR	NR	73.1	756	26	Low
Ceballos	2021	Chile	16 April and 23 June 2020	23 hospitals all over the country	Multi-Site study	HR	HB	44 (IQR: 26–85)	NR	NR	HIV positive: 92	18,321	36	Low
Charre	2020	France	March to April 2020	Lyon	Unclear/Not described	Yes	PB	HIV positive: 53.0 (41.3–58.6), HIV negative:54.6 (35.6–75.7)	NR	NR	HIV positive:67.5, HIV negative:40.5	3648	12	Low
Collins	2020	USA	8 March 2020 to 23 April 2020	Atlanta	Multi-Site study	HR	HB	57 (IQR: 48–62)	NR	NR	HIV positive: 65	530	20	Low
Cucurull-Canosa	2021	Spain	Up to 15 May 2020	Madrid	Single-Site study	HR	HB	HIV:22.7	HIV:4	HIV:35	HIV:58.3	317	12	Low
Díez	2021	Spain	Up to 30 June 2020	13 hospitals of the 17 regions of the country	Multi-Site study	HR	HB	HIV:53; HIV negative:53	Q1: HIV:46; HIV negative: 46	Q3: HIV:56; HIV negative:56	HIV:90.5; HIV negative: 90.5	126	21	Low
Durstenfeld	2021	USA	Up to December 2020	107 hospitals in USA	Multi-Site study	Yes	HB	HIV: 56.0 ± 13.0; HIV negative: 62.3 ± 17.9	NR	NR	HIV:72.3; HIV negative:53.9	21,528	220	Low
Esfahanian	2021	Iran	From 20 February to 19 April 2020	Tehran	Multi-Site study	HR	HB	NR	NR	NR	66.4	500	4	Low
Geretti	2020	England, Scotland, and Wales	June 2020	Multi-countries	Multi-Site study	Yes	HB	HIV positive: 56 (IQR:49, 62) HIV negative: 74 (60, 84)	NR	NR	HIV positive:66.1, HIV negative: 57.1	47,592	122	Low
Gudipati	2020	USA	March 20, 2020, and April 30, 2020	Michigan	Multi-Site study	HR	HB	HIV positive:49, HIV negative:52	NR	NR	HIV positive: 81; HIV negative:47	65,549	278	Low
Hadi	2020	USA	NR	Massachusetts	Multi-Site study	Yes	HB	HIV positive:48.2; HIV negative:48.8	10	NR	HIV positive: 70.6; HIV negative:44.9	50,167	404	Low
Jassat	2021	South Africa	Up to March 27, 2021	393 public and 251 private hospitals	Multi-Site study	Yes	HB	NR	NR	NR	HIV:7.2; HIV negative:92.8	151,779	13,793	Low
Lee	2021	UK	From 1 February 2020 to 31 May 2020	London, Manchester, and Leicester	Multi-Site study	HR	HB	HIV:57; HIV negative:56	Q1: HIV: 50; HIV negative: 51	Q3: HIV: 63; HIV negative:62	HIV:61.8; HIV negative: 63	249	68	Low
Molina-Iturritza	2020	Spain	1 March to 30 April 2020	Araba	Multi-Site study	HR	HB	NR	NR	NR	HIV positive:78, HIV negative:55	8912	161	Low
Mwananyanda	2021	Zambia	Up to september 2020	Lusaka	Single-Site study	HR	HB	48	Q1:36	Q3:72	69	70	16	Low
Karmen-Tuohy	2020	USA	March 2, 2020, and April 23, 2020	New York	Multi-Site study	Yes	HB	NR	NR	NR	NR	NA	NA	Low
Kirenga	2020	Uganda	16 May 2020	Entebbe	Multi-Site study	HR	HB	34.2	NR	NR	67.9	203	15	Low
Mash	2021	South Africa	March and June 2020	Western Cape	Multi-Site study	HR	HB	46.3	NR	NR	NR	1376	195	Low
Mbarga	2021	Cameroon	April,01, 2020 to July,31, 2020	Yaoundé	Single-Site study	HR	HB	46	NR	NR	62.5	259	7	Low
Migisha	2020	Uganda	March 21–April 12, 2020	National	Multi-Site study	Yes	PB	35	NR	NR	63	54	2	Low
Tshikung	2021	Switzerland	1 May 2020	Geneva	Single-Site study	HR	HB	NR	NR	NR	NR	1024	8	Low
Venturas	2021	South Africa	March 6, 2021, to September 11, 2020	Johannesburg	Single-Site study	HR	HB	HIV: 45; HIV negative: 52.5	Q1: HIV: 38; HIV negative:39.8	Q3: HIV: 56; HIV negative: 61	HIV: 50; HIV negative:54	384	108	Low
Nagarakanti	2021	Israel	March 2020 and April 2020	Newark Beth	Single-Site study	HR	HB	HIV: 59; HIV negative: 49	HIV: 51; HIV negative: 41	HIV: 67; HIV negative: 73	HIV: 61.0; HIV negative:34.8	66	23	Low
Silva	2020	Portugal	March 02 and July 14, 2020	Porto	Single-Site study	HR	HB	48	NR	NR	NR	2092	8	Low
Sultan	2021	Ethiopia	Up to August 20, 2020	Addis Ababa	Multi-Site study	HR	HB	59	17	92	71	85	15	Low
Sun	2021	USA	Up to 21 May 2021	National	Multi-Site study	Yes	HB	HIV: 50; HIV negative:47	Q1: HIV: 36; HIV negative:32	Q3: HIV: 59; HIV negative:61	HIV: 44.8; HIV negative:72.5	1,446,913	8270	Low
Wyk	2020	South Africa	3 July 2020	National	Multi-Site study	Yes	PB	61	NR	NR	52.0	2457	342	Low
Yang	2020	China	February 14	Wuhan	Multi-Site study	HR	HB	NR	NR	NR	NR	188	3	Low
Shalev	2020	USA	15 April 2020	New York	Single-Site study	Yes	HB	60.7	23	89	NR	2159	31	Low
Ouyang	2020	USA	3/1 to 5/15, 2020	New York	Single-Site study	HR	HB	NR	NR	NR	NR	1092	22	Low
Parker	2020	South Africa	25 March to 11 May 2020	Cape Town	Single-Site study	HR	HB	HIV positive: 46.2; HIV negative: 49.1	NR	NR	HIV positive: 25.0; HIV negative: 42.7	116	24	Low
Rosenthal	2020	USA	April 1 and May 31, 2020	National	Multi-Site study	Yes	HB	55.5	NR	NR	49.3	64,781	252	Low
Sachdev	2021	USA	March 24, 2020, to September 3, 2020	San Francisco	Multi-Site study	Yes	PB	48	13	NR	91.2	9819	193	Low
Sigle	2020	USA	12 March and 23 April 2020	Mount Sinai	Multi-Site study	Yes	HB	HIV positive: 61; HIV negative: 60	NR	NR	HIV positive: 75, HIV negative: 76	4402	88	Low
Stoeckle	2020	USA	March 3, 2020, and May 15, 2020	New York	Single-Site study	HR	HB	HIV positive: 60.5; HIV negative: 60.5	NR	NR	HIV positive: 80, HIV negative: 80	NA	NA	Low
Tesoriero	2021	USA	March 1 and June 15, 2020,	New York	Multi-Site study	Yes	HB	54.0	NR	NR	Unclear	19,453,561	2409	Low
Yendewa	2021	USA	January 1 to December 1, 2020	44 healthcare centers in the USA	Multi-Site study	Yes	HB	HIV:48.34 ± 13.59; HIV negative:48.34 ± 13.59	NR	NR	HIV:69.4; HIV negative: 69.4	297,194	1638	Low

HB: Hospital-based; PB: population-based; HR: hospital records; Max age: maximum age; Min age: minimum age; RoB: risk of bias

### Prevalence of HIV among patients with COVID-19

**Fig. 1:**
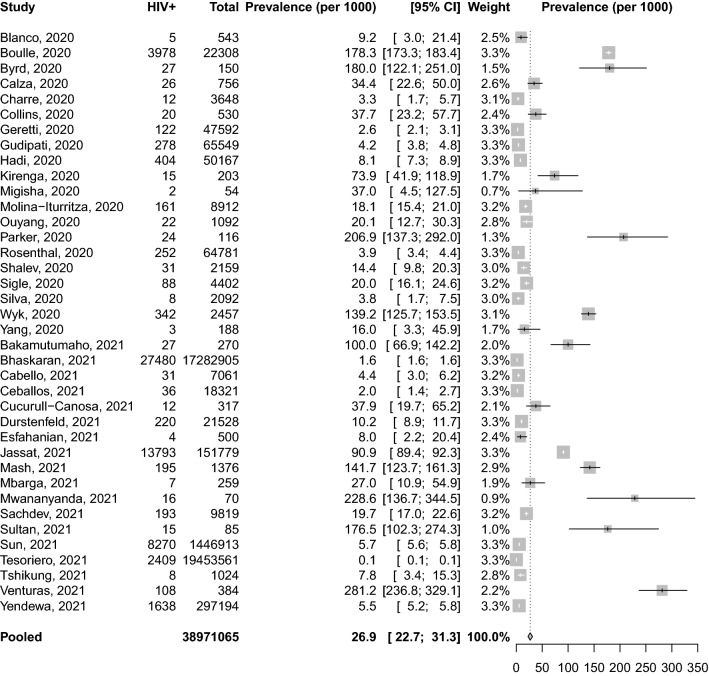
Proportion of HIV positive patients among patients with COVID-19

Thirty-eight studies were included in the meta-analysis of prevalence information. The pooled prevalence of HIV among COVID-19 patients was 26.9 ‰ (95% confidence interval [CI] 22.7–31.3) (Fig. 1) and was significantly higher in studies conducted in Africa compared to those conducted elsewhere (118.5‰ [95% CI 84.8–156.9, 11 studies] vs 10.9‰ [95% CI 8.8–13.2, 27 studies]). The pooled prevalence of HIV among COVID-19 patients was 12.9‰ (95% CI 7.7–19.5, 13 studies) in the USA and was significantly lower compare with the figure outside the USA (49.2‰; 95% CI 24.0–82.2, 25 studies) (P value: 0.002). The pooled prevalence of HIV among studies conducted on hospital records was 24.6‰ (95% CI 20.4–29.1, 33 studies), while the figure for population-based studies was 56.8‰ (95% CI 11.9–129.7, 5 studies) (Additional file 1: Figs. S1–S4).

### Risk of in-hospital admission associated with HIV infection

**Fig. 2:**
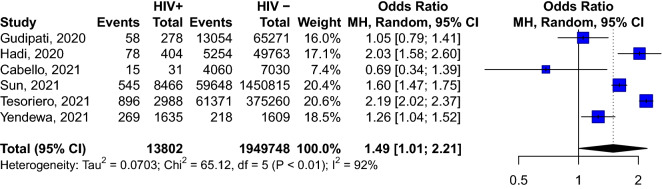
Risk of hospital admission according to HIV status among COVID-19 patients

Based on the meta-analysis of six studies, HIV-positive COVID-19 participants were more likely to be admitted to hospital than HIV-negative patients (OR: 1.49; 95% CI 1.01–2.21) (Fig. 2).

### Risk of severe COVID-19 associated with HIV infection

**Fig. 3:**
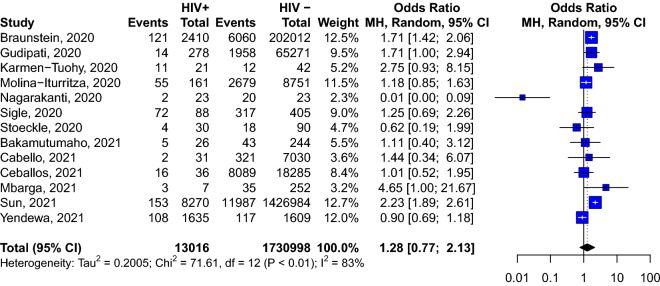
Risk of presenting severe COVID-19 infection according to HIV status

A meta-analysis of 13 studies including 13,016 HIV-infected individuals with COVID-19, and 1,744,014 HIV-uninfected individuals with COVID-19, shows that HIV does not increase the likelihood of having severe COVID-19 (OR: 1.28; 95% CI 0.77–2.13) (Fig. 3), even after stratification according to study’s country of recruitment (Additional file 1: Figs. S5, S6).

### Risk of death from COVID-19 associated with HIV infection

Twenty-three studies were included in the unadjusted risk ratio meta-analysis and two (one from South Africa, and one multicentric) in the adjusted HR meta-analysis.

**Fig. 4:**
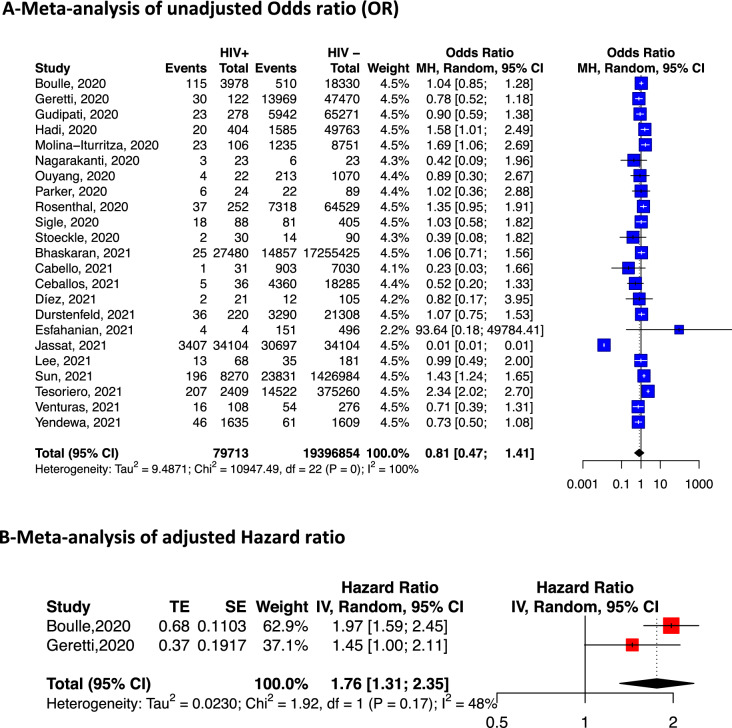
Risk of death among COVID-19 patients according to HIV status. Meta-analysis of adjusted and unadjusted estimates

In unadjusted pooled analyses, there was no association between death from COVID-19 and HIV (OR: 0.81; 95% CI 0.47; 1.41, 23 studies). However, in analyses adjusted for age and sex, HIV was associated with an increased risk of death (hazard ratio 1.76, 95% CI 1.31–2.35; 2 studies) (Fig. 4).

There was no significant difference in the risk of death of patients co-infected with HIV and COVID-19, compare with those without HIV even when the analysis was stratified by country (Additional file 1: Figs. S7, S8).

### Publication bias and sensitivity analysis

The funnel plot of the studies included in the prevalence meta-analysis shows some asymmetry which was confirmed by Egger's test (p-value = 0.01) (Additional file 1: Fig. S9), suggesting the presence of publication bias. However, neither the funnel plot nor the Egger test indicated publication bias for studies included in the meta-analysis conducted to assess the risk of in-hospital admission, severe disease, or death (Additional file 1: Figs. S10–S12).

In the leave-one-out analysis, none of the studies included when omitted change the overall effect-size in all analyses except in the meta-analysis pertaining to mortality risk (Additional file 1: Figs. S13–S16). In the latter, the omission of the Jassap et al. study strongly influences the overall OR, without changing the direction of the association between HIV and COVID-19 mortality, which remains non-significant (Additional file 1: Fig. S16).

## Discussion

The results of the current study suggest that co-infection with HIV is associated with an increased risk of hospital admission in people with COVID-19. Furthermore, based on the analysis of a limit number of studies, the meta-analysis of adjusted (for age and sex) hazard ratio showed that HIV increases the risk of death in patients with COVID-19. However, HIV was not associated with an increased risk of death or of developing severe disease in the unadjusted analysis. The influence of age and sex on the outcome of patients with COVID-19 is well known and has been previously published [31, 32]. The lack of evidence of higher risk of death in HIV patients with COVID-19 in previous meta-analyses on the topic is probably because these meta-analyses pooled unadjusted estimators [14, 33].

Indeed, in our study, when pooling the unadjusted risk ratios, no difference in terms of mortality is observed. This contradictory result draws attention on the need to consider homogeneously adjusted estimators in the meta-analyses rather than raw estimators [11]. The possibility that the effect observed in the unadjusted analysis is attributable to sex cannot be excluded, as sex is known to influence the outcome of COVID-19 patients [34–40]. Our findings could have been different if the analyses had been stratified according to CD4 count or ARV protocol. Indeed, some ARVs such as tenofovir disoproxil fumarate/emtricitabine (TDF/FTC) are reported to be potentially effective against COVID-19 and could have a protective effect in patients on these drugs, thus modifying the natural history of the disease in patients treated with these medications [41–44]. In addition, CD4 count and lymphopenia, are known to be associated with disease severity and could have an impact on the evolution of COVID-19, as one of the main mechanisms underlying COVID-19-related morbidity and mortality is cytokine storm [45]. Deep immunodepression and low CD4 count could therefore increase the probability of having lymphopenia and a pejorative course of COVID-19 in HIV patients. Indeed in two recent studies presented in the conference on retrovirus and opportunistic infections (CROI), the mortality rate between HIV-positive and HIV-negatives patients with COVID-19 was not statistically different in treated and well- controlled patients [46, 47].

The relationship between age and increased comorbidities in HIV-positive patients compared to their negative counterparts is well established. The presence of inflammation in patients with HIV, even under effective ART, is thought to be the cause of renal, cardiovascular, and neurological diseases [48, 49]. These comorbidities are associated with poor outcomes of COVID-19 [44, 49].

Our results point to a potential increased risk of admission for COVID-19 in HIV-infected individuals. This probably reflects the conservative approach used by physicians for this category of patients, given the inconsistent evidence regarding their outcome. Indeed, knowing the vulnerability of HIV patients to infections due to the pathophysiology of the disease [43], physicians may be inclined to admit HIV-positive individuals more easily than HIV-uninfected individuals, in order to better monitor them in hospital and anticipate the occurrence of any potential complications.

Our results also shows that co-infection with HIV does not increase the risk of presenting severe forms of COVID-19 as previously found by other authors [12]. Several hypotheses have been suggested to explain this phenomenon. The most plausible of which is the presence of immunodepression, which prevents patients from triggering and maintaining the cytokine storm responsible for the inflammatory manifestations of the disease, and which intensity is correlated with the severity of the disease. However, this claim would only be valid in HIV-immunocompromised patients with low CD4 counts and high viral load. A meta-analysis stratifying the outcomes according to CD4 count would therefore make it possible to distinguish whether severely immunocompromised patients are less likely to present severe COVID-19 compare with patients on ARVs with a CD4 count above 200 cells/mm^3^. This especially because some studies have shown a worser prognosis in patients with CD4 counts below 200/mm^3^ [48]. Unfortunately, few studies included in our meta-analysis stratified their results according to CD4 count, making it difficult to pooled studies according to CD4 count and to assess the veracity of this hypothesis using available evidence.

The current study highlights the need to consider HIV patients as a sub-population at high risk of hospital admission. They also call for more studies stratifying their analyses according to the different conditions (gender, age) and comorbidities known to influence the course of COVID-19, to clarify the contribution of HIV in disease progression.

Several reviews have attempted to synthesise outcome information for HIV patients with COVID-19 [12–17, 50]. However, these studies have either included preprints and therefore unpublished articles in peer-reviewed journals, or they have meta-analysed unadjusted estimators, ignoring the potential difference in the composition of the study populations and, more importantly, the presence of factors such as co-morbidities other than HIV that could influence outcomes in primary studies. The strength of our study was to give for the first time a meta-analysis of adjusted estimators and to included only articles published in peer review journals. Furthermore, the pooled sample size in our meta-analysis was high (38,971,065 patients with COVID-19). However, some limitations of the current study are the absence of the stratification of the analysis according to ART regimen, and the level of CD4. This was due to the lack of sufficient information to conduct these subgroup analyses. Secondly the number of studies included in the meta-analysis of adjusted point estimates was low.

## Conclusion

Findings of the current review suggest that patients with HIV have an increased risk of hospital admission. Although crude analysis did not show an association between HIV infection and an increased risk of death or of developing severe disease in patients with COVID-19, adjusted data from two studies suggest that HIV infection increased the risk of mortality due to COVID-19. However, this later evidence was weak as it was derived from only two studies.

## Supplementary Information

**Additional file 1.** Additional Figures and Tables.

## Data Availability

All materials are available in the manuscript and additional file.
