# Supplementary material for: Outcomes of patients with HIV and COVID-19 co-infection: a systematic review and meta-analysis
Source: AIDS Res Ther. 2022 Jan 14;19:3. doi: 10.1186/s12981-021-00427-y (PMC8759058; doi:10.1186/s12981-021-00427-y)
Supplement: Supplementary file 1 — Additional file 1. Additional Figures and Tables. [file 12981_2021_427_MOESM1_ESM.pdf]

Outcomes of patients with HIV and COVID-19 co-infection: a systematic review and meta-analysis

APPENDIX

Supplementary Figures

Figure 1. PRISMA flow 2
Figure 2.Proportion of HIV patients among COVID-19 patients according to location (Africa vs Outside Africa) 4
Figure 3. Proportion of HIV patients among COVID-19 patients according to location (USA vs Outside USA) 5
Figure 4. Proportion of HIV patients among COVID-19 patients according to type of study 6
Figure 5. Odd of presenting with severe COVID-19 according to country location (USA vs Outside USA) 7
Figure 6. Odd of presenting with severe COVID-19 according to country location (Africa vs Outside Africa) 7
Figure 7. Mortality risk according to country location (USA vs Outside USA) 8
Figure 8. Mortality risk according to country location (Africa vs Outside Africa) 9
Figure 9. Funnel plot of studies included in the meta-analysis of prevalence 9
Figure 10. Funnel plot of studies included in the meta-analysis of In-hospital admission 10
Figure 11. Funnel plot of studies included in the meta-analysis of severity assessment 10
Figure 12. Funnel plot of studies included in the meta-analysis of mortality 11
Figure 13. sensitivity analysis of studies included in prevalence assessment meta-analysis 11
Figure 14. sensitivity analysis of studies included in in-hospital admission risk meta-analysis 12
Figure 15. sensitivity analysis of studies included in Severity assessment meta-analysis 12
Figure 16. sensitivity analysis of studies included in mortality risk assessment 12

Supplementary Tables

Table 1. Search strategy for PubMed 3
Table 2. Search strategy for EMBASE 3
Table 3. Search strategy for SCOPUS 3

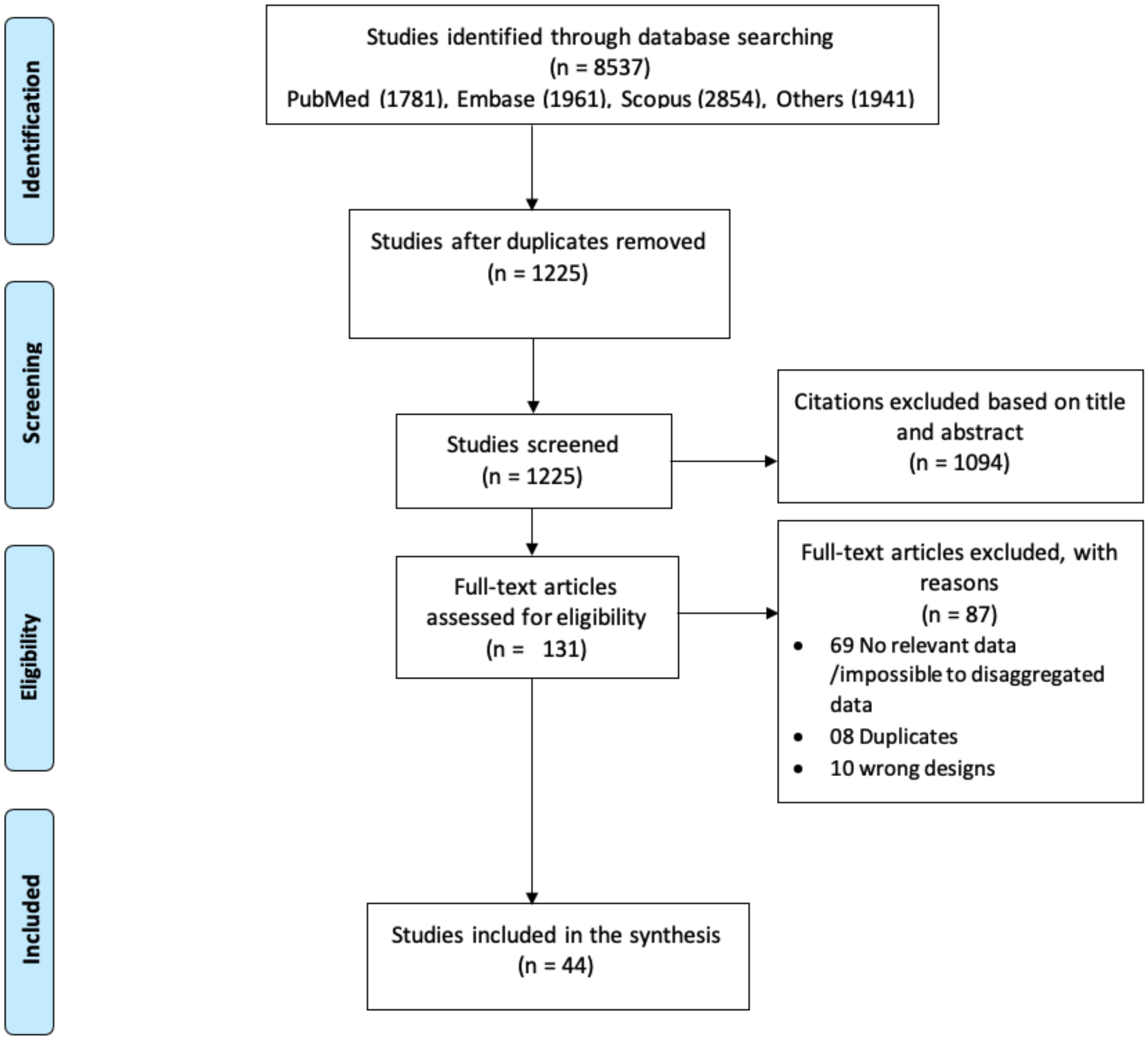

Figure 1. PRISMA flow

Table 1. Search strategy for PubMed

Search	Search terms
#1	"HIV"[MeSH] OR "HIV"[tiab]
#2	"COVID-19"[MeSH Terms] OR "COVID-19"[tiab]
#3	#1 AND #2
Date: 25 October 2021: 1781 citations found	

Table 2. Search strategy for EMBASE

Search	Search terms
#1	'human immunodeficiency virus infection':ti,ab,kw OR hiv:ti,ab,kw
#2	'coronavirus disease 2019':ti,ab,kw OR 'covid 19':ti,ab,kw OR 'sars cov2':ti,ab,kw
#3	#1 AND #2
Date: 25 October 2021: 1961 citations found	

Table 3. Search strategy for SCOPUS

Search	Search terms
#1	(TITLE-ABS-KEY-AUTH (*hiv*) OR TITLE-ABS-KEY ('*human* AND *immunodeficiency* AND *virus* AND *infection*'))
#2	(TITLE-ABS-KEY-AUTH ('*coronavirus* AND *disease* AND *2019*') OR TITLE-ABS-KEY ('*covid* AND *19*') OR TITLE-ABS-KEY (*sars* AND *cov2*))
#3	#1 AND #2
Date: 25 October 2021: 2854 citations found	

Proportion of HIV patients among COVID-19 patients according to location (Africa vs Outside Africa)

Study	HIV+	Total	Prevalence,‰	[95% CI]	Weight
**Africa**					
Boulle, 2020	3978	22308	178.3	[173.3; 183.4]	3.3%
Kirenga, 2020	15	203	73.9	[41.9; 118.9]	1.7%
Migisha, 2020	2	54	37.0	[4.5; 127.5]	0.7%
Parker, 2020	24	116	206.9	[137.3; 292.0]	1.3%
Wyk, 2020	342	2457	139.2	[125.7; 153.5]	3.1%
Bakamutumaho, 2021	27	270	100.0	[66.9; 142.2]	2.0%
Jassat, 2021	13793	151779	90.9	[89.4; 92.3]	3.3%
Mash, 2021	195	1376	141.7	[123.7; 161.3]	2.9%
Mbarga, 2021	7	259	27.0	[10.9; 54.9]	1.9%
Mwananyanda, 2021	16	70	228.6	[136.7; 344.5]	0.9%
Sultan, 2021	15	85	176.5	[102.3; 274.3]	1.0%
**Subgroup Prevalence**		**178977**	**118.5**	**[84.8; 156.9]**	**22.1%**
Heterogeneity: $I^2 = 99.3\%$, $p < 0.0001$					
**Outside Africa**					
Blanco, 2020	5	543	9.2	[3.0; 21.4]	2.5%
Byrd, 2020	27	150	180.0	[122.1; 251.0]	1.5%
Calza, 2020	26	756	34.4	[22.6; 50.0]	2.6%
Charre, 2020	12	3648	3.3	[1.7; 5.7]	3.1%
Collins, 2020	20	530	37.7	[23.2; 57.7]	2.4%
Geretti, 2020	122	47592	2.6	[2.1; 3.1]	3.3%
Gudipati, 2020	278	65549	4.2	[3.8; 4.8]	3.3%
Hadi, 2020	404	50167	8.1	[7.3; 8.9]	3.3%
Molina-Iturritza, 2020	161	8912	18.1	[15.4; 21.0]	3.2%
Ouyang, 2020	22	1092	20.1	[12.7; 30.3]	2.8%
Rosenthal, 2020	252	64781	3.9	[3.4; 4.4]	3.3%
Shalev, 2020	31	2159	14.4	[9.8; 20.3]	3.0%
Sigle, 2020	88	4402	20.0	[16.1; 24.6]	3.2%
Silva, 2020	8	2092	3.8	[1.7; 7.5]	3.0%
Yang, 2020	3	188	16.0	[3.3; 45.9]	1.7%
Bhaskaran, 2021	27480	17282905	1.6	[1.6; 1.6]	3.3%
Cabello, 2021	31	7061	4.4	[3.0; 6.2]	3.2%
Ceballos, 2021	36	18321	2.0	[1.4; 2.7]	3.3%
Cucurull-Canosa, 2021	12	317	37.9	[19.7; 65.2]	2.1%
Durstenfeld, 2021	220	21528	10.2	[8.9; 11.7]	3.3%
Esfahanian, 2021	4	500	8.0	[2.2; 20.4]	2.4%
Sachdev, 2021	193	9819	19.7	[17.0; 22.6]	3.2%
Sun, 2021	8270	1446913	5.7	[5.6; 5.8]	3.3%
Tesoriero, 2021	2409	19453561	0.1	[0.1; 0.1]	3.3%
Tshikung, 2021	8	1024	7.8	[3.4; 15.3]	2.8%
Venturas, 2021	108	384	281.2	[236.8; 329.1]	2.2%
Yendewa, 2021	1638	297194	5.5	[5.2; 5.8]	3.3%
**Subgroup Prevalence**		**38792088**	**10.9**	**[8.8; 13.2]**	**77.9%**
Heterogeneity: $I^2 = 99.9\%$, $p = 0$					
**Pooled**		**38971065**	**26.9**	**[22.7; 31.3]**	**100.0%**
Test for subgroup differences: $\chi^2_1 = 75.07$, df = 1 ($p < 0.0001$)					

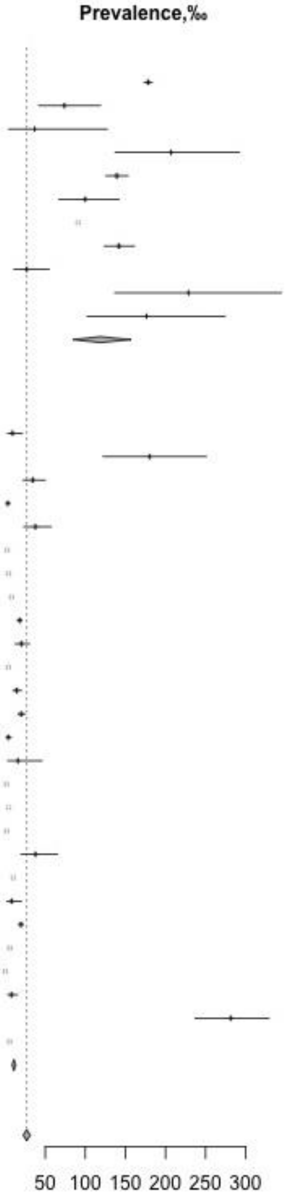

Figure 2.Proportion of HIV patients among COVID-19 patients according to location (Africa vs Outside Africa)

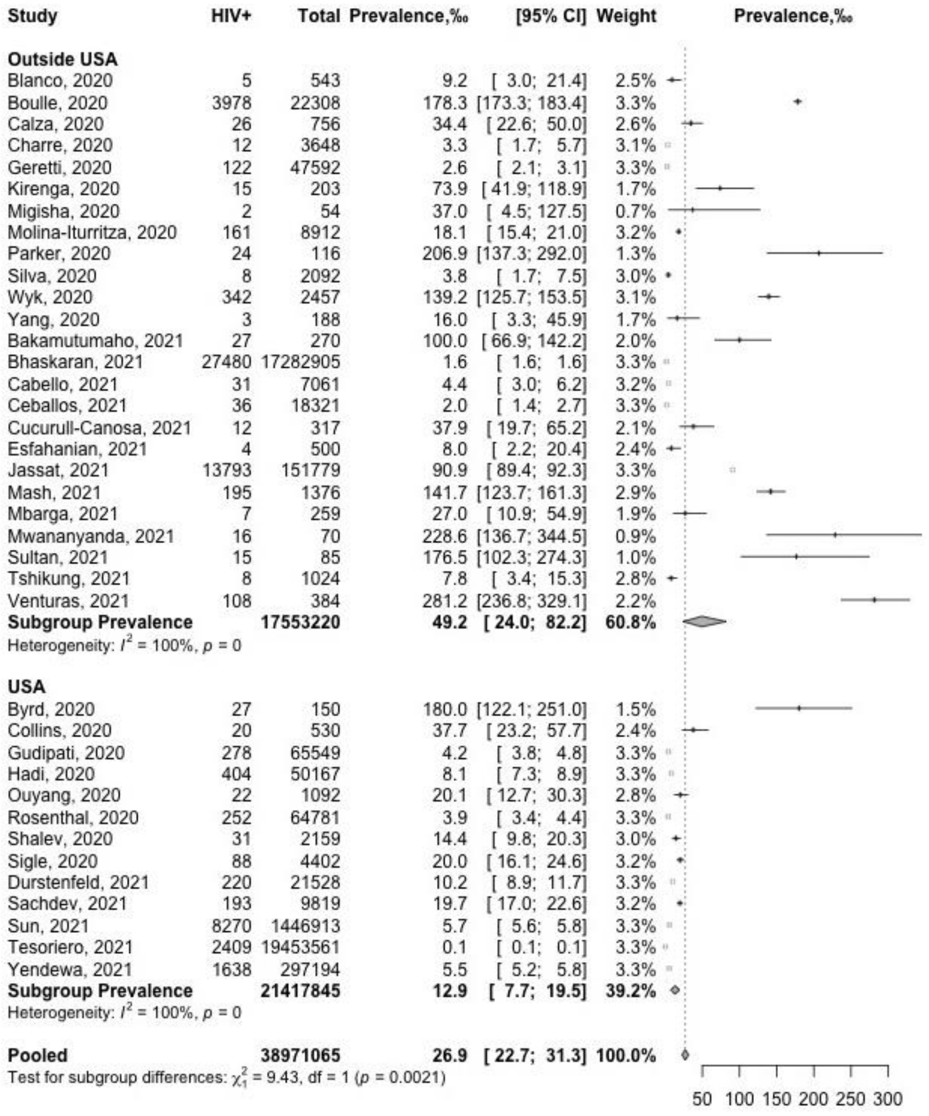

Figure 3. Proportion of HIV patients among COVID-19 patients according to location (USA vs Outside USA)

Study	HIV+	Total	Prevalence,‰	[95% CI]	Weight
**HB**					
Blanco, 2020	5	543	9.2	[3.0; 21.4]	2.5%
Boulle, 2020	3978	22308	178.3	[173.3; 183.4]	3.3%
Calza, 2020	26	756	34.4	[22.6; 50.0]	2.6%
Collins, 2020	20	530	37.7	[23.2; 57.7]	2.4%
Geretti, 2020	122	47592	2.6	[2.1; 3.1]	3.3%
Gudipati, 2020	278	65549	4.2	[3.8; 4.8]	3.3%
Hadi, 2020	404	50167	8.1	[7.3; 8.9]	3.3%
Kirenga, 2020	15	203	73.9	[41.9; 118.9]	1.7%
Molina-Iturritza, 2020	161	8912	18.1	[15.4; 21.0]	3.2%
Ouyang, 2020	22	1092	20.1	[12.7; 30.3]	2.8%
Parker, 2020	24	116	206.9	[137.3; 292.0]	1.3%
Rosenthal, 2020	252	64781	3.9	[3.4; 4.4]	3.3%
Shalev, 2020	31	2159	14.4	[9.8; 20.3]	3.0%
Sigle, 2020	88	4402	20.0	[16.1; 24.6]	3.2%
Silva, 2020	8	2092	3.8	[1.7; 7.5]	3.0%
Yang, 2020	3	188	16.0	[3.3; 45.9]	1.7%
Bakamutumaho, 2021	27	270	100.0	[66.9; 142.2]	2.0%
Bhaskaran, 2021	27480	17282905	1.6	[1.6; 1.6]	3.3%
Cabello, 2021	31	7061	4.4	[3.0; 6.2]	3.2%
Ceballos, 2021	36	18321	2.0	[1.4; 2.7]	3.3%
Cucurull-Canosa, 2021	12	317	37.9	[19.7; 65.2]	2.1%
Durstenfeld, 2021	220	21528	10.2	[8.9; 11.7]	3.3%
Esfahanian, 2021	4	500	8.0	[2.2; 20.4]	2.4%
Jassat, 2021	13793	151779	90.9	[89.4; 92.3]	3.3%
Mash, 2021	195	1376	141.7	[123.7; 161.3]	2.9%
Mbarga, 2021	7	259	27.0	[10.9; 54.9]	1.9%
Mwananyanda, 2021	16	70	228.6	[136.7; 344.5]	0.9%
Sultan, 2021	15	85	176.5	[102.3; 274.3]	1.0%
Sun, 2021	8270	1446913	5.7	[5.6; 5.8]	3.3%
Tesoriero, 2021	2409	19453561	0.1	[0.1; 0.1]	3.3%
Tshikung, 2021	8	1024	7.8	[3.4; 15.3]	2.8%
Venturas, 2021	108	384	281.2	[236.8; 329.1]	2.2%
Yendewa, 2021	1638	297194	5.5	[5.2; 5.8]	3.3%
**Subgroup Prevalence**		**38954937**	**24.6**	**[20.4; 29.1]**	**88.3%**
Heterogeneity: I^2 = 100%, p = 0					
**PB**					
Byrd, 2020	27	150	180.0	[122.1; 251.0]	1.5%
Charre, 2020	12	3648	3.3	[1.7; 5.7]	3.1%
Migisha, 2020	2	54	37.0	[4.5; 127.5]	0.7%
Wyk, 2020	342	2457	139.2	[125.7; 153.5]	3.1%
Sachdev, 2021	193	9819	19.7	[17.0; 22.6]	3.2%
**Subgroup Prevalence**		**16128**	**56.8**	**[11.9; 129.7]**	**11.7%**
Heterogeneity: I^2 = 99.4%, p < 0.0001					
**Pooled**		**38971065**	**26.9**	**[22.7; 31.3]**	**100.0%**
Test for subgroup differences: χ^2_1 = 1.73, df = 1 (p = 0.1880)					

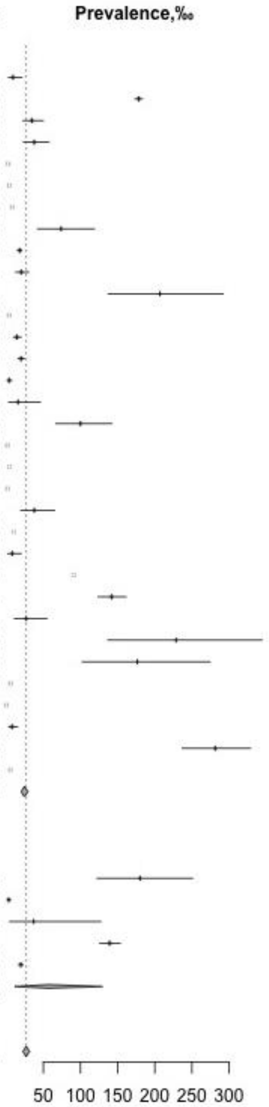

HB: hospital-based, PB: Population-based

Figure 4. Proportion of HIV patients among COVID-19 patients according to type of study

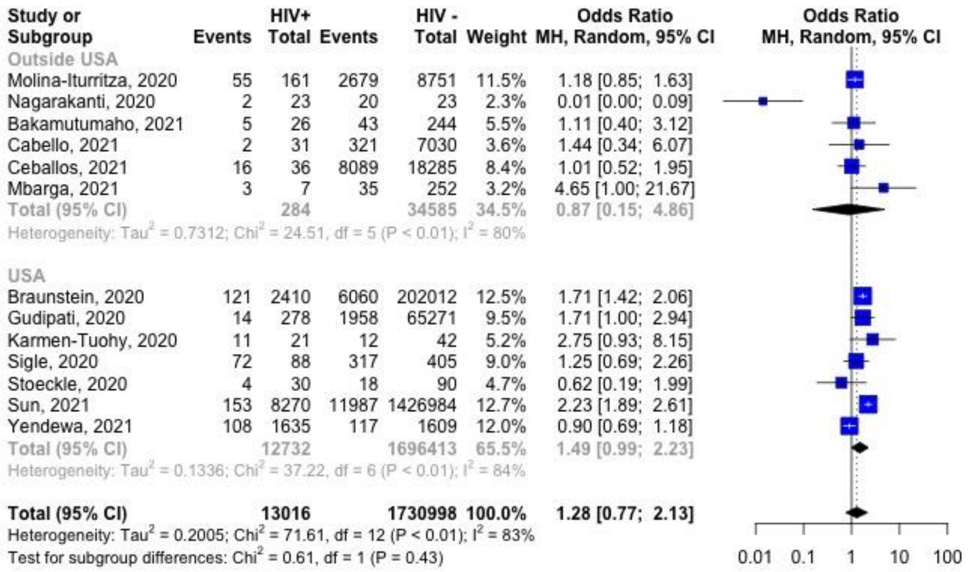

Figure 5. Odd of presenting with severe COVID-19 according to country location (USA vs Outside USA)

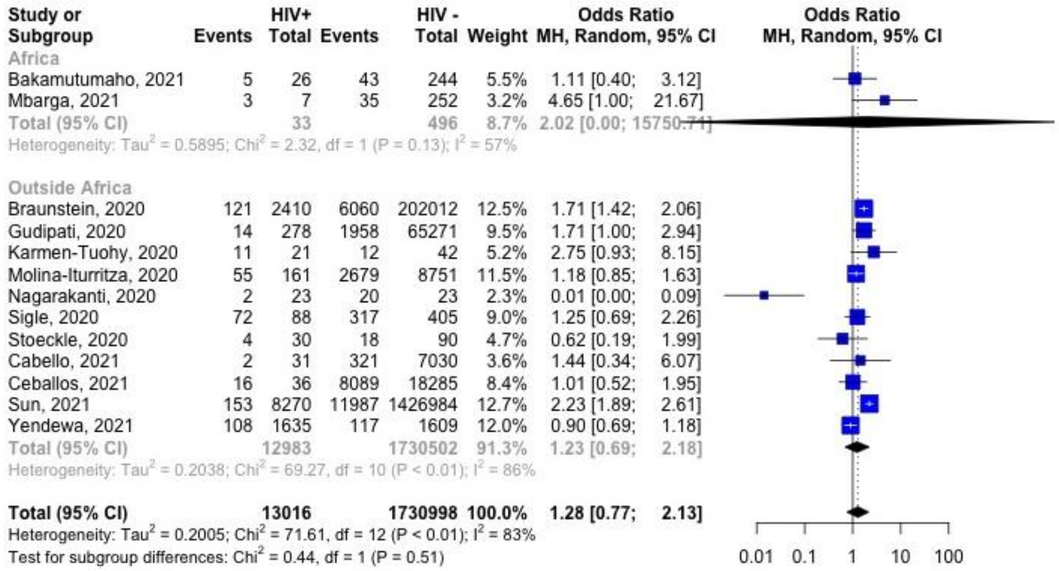

Figure 6. Odd of presenting with severe COVID-19 according to country location (Africa vs Outside Africa)

Study or Subgroup	HIV+ Events	HIV+ Total	HIV - Events	HIV - Total	Weight	Odds Ratio MH, Random, 95% CI
Outside USA						
Boulle, 2020	115	3978	510	18330	4.5%	1.04 [0.85; 1.28]
Geretti, 2020	30	122	13969	47470	4.5%	0.78 [0.52; 1.18]
Molina-Iturritza, 2020	23	106	1235	8751	4.5%	1.69 [1.06; 2.69]
Nagarakanti, 2020	3	23	6	23	4.3%	0.42 [0.09; 1.96]
Parker, 2020	6	24	22	89	4.4%	1.02 [0.36; 2.88]
Bhaskaran, 2021	25	27480	14857	17255425	4.5%	1.06 [0.71; 1.56]
Cabello, 2021	1	31	903	7030	4.1%	0.23 [0.03; 1.66]
Ceballos, 2021	5	36	4360	18285	4.4%	0.52 [0.20; 1.33]
Díez, 2021	2	21	12	105	4.2%	0.82 [0.17; 3.95]
Esfahanian, 2021	4	4	151	496	2.2%	93.64 [0.18; 49784.41]
Jassat, 2021	3407	34104	30697	34104	4.5%	0.01 [0.01; 0.01]
Lee, 2021	13	68	35	181	4.5%	0.99 [0.49; 2.00]
Venturas, 2021	16	108	54	276	4.5%	0.71 [0.39; 1.31]
Total (95% CI)		66105		17390565	55.2%	0.65 [0.24; 1.78]
Heterogeneity: $Tau^2 = 9.5813$; $Chi^2 = 3336.37$, df = 12 (P = 0); $I^2 = 100\%$						
USA						
Gudipati, 2020	23	278	5942	65271	4.5%	0.90 [0.59; 1.38]
Hadi, 2020	20	404	1585	49763	4.5%	1.58 [1.01; 2.49]
Ouyang, 2020	4	22	213	1070	4.4%	0.89 [0.30; 2.67]
Rosenthal, 2020	37	252	7318	64529	4.5%	1.35 [0.95; 1.91]
Sigle, 2020	18	88	81	405	4.5%	1.03 [0.58; 1.82]
Stoeckle, 2020	2	30	14	90	4.3%	0.39 [0.08; 1.82]
Durstenfeld, 2021	36	220	3290	21308	4.5%	1.07 [0.75; 1.53]
Sun, 2021	196	8270	23831	1426984	4.5%	1.43 [1.24; 1.65]
Tesoriero, 2021	207	2409	14522	375260	4.5%	2.34 [2.02; 2.70]
Yendewa, 2021	46	1635	61	1609	4.5%	0.73 [0.50; 1.08]
Total (95% CI)		13608		2006289	44.8%	1.20 [0.89; 1.61]
Heterogeneity: $Tau^2 = 0.1484$; $Chi^2 = 64.83$, df = 9 (P < 0.01); $I^2 = 86\%$						
**Total (95% CI)**		**79713**		**19396854**	**100.0%**	**0.81 [0.47; 1.41]**

Heterogeneity: $Tau^2 = 9.4871$; $Chi^2 = 10947.49$, df = 22 (P = 0); $I^2 = 100\%$

Test for subgroup differences: $Chi^2 = 1.61$, df = 1 (P = 0.20)

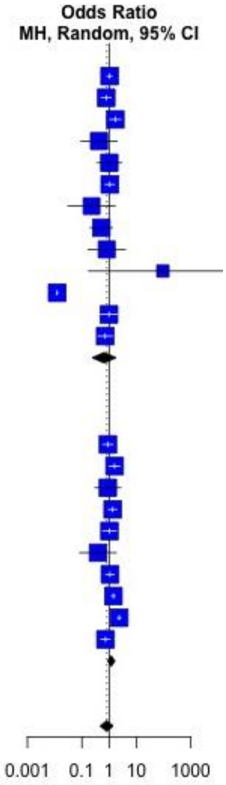

Figure 7. Mortality risk according to country location (USA vs Outside USA)

Study or Subgroup	HIV+ Events	HIV+ Total	HIV - Events	HIV - Total	Weight	Odds Ratio MH, Random, 95% CI
**Outside Africa**						
Geretti, 2020	30	122	13969	47470	4.5%	0.78 [0.52; 1.18]
Gudipati, 2020	23	278	5942	65271	4.5%	0.90 [0.59; 1.38]
Hadi, 2020	20	404	1585	49763	4.5%	1.58 [1.01; 2.49]
Molina-Iturritza, 2020	23	106	1235	8751	4.5%	1.69 [1.06; 2.69]
Nagarakanti, 2020	3	23	6	23	4.3%	0.42 [0.09; 1.96]
Ouyang, 2020	4	22	213	1070	4.4%	0.89 [0.30; 2.67]
Rosenthal, 2020	37	252	7318	64529	4.5%	1.35 [0.95; 1.91]
Sigle, 2020	18	88	81	405	4.5%	1.03 [0.58; 1.82]
Stoeckle, 2020	2	30	14	90	4.3%	0.39 [0.08; 1.82]
Bhaskaran, 2021	25	27480	14857	17255425	4.5%	1.06 [0.71; 1.56]
Cabello, 2021	1	31	903	7030	4.1%	0.23 [0.03; 1.66]
Ceballos, 2021	5	36	4360	18285	4.4%	0.52 [0.20; 1.33]
Díez, 2021	2	21	12	105	4.2%	0.82 [0.17; 3.95]
Durstenfeld, 2021	36	220	3290	21308	4.5%	1.07 [0.75; 1.53]
Esfahanian, 2021	4	4	151	496	2.2%	93.64 [0.18; 49784.41]
Lee, 2021	13	68	35	181	4.5%	0.99 [0.49; 2.00]
Sun, 2021	196	8270	23831	1426984	4.5%	1.43 [1.24; 1.65]
Tesoriero, 2021	207	2409	14522	375260	4.5%	2.34 [2.02; 2.70]
Venturas, 2021	16	108	54	276	4.5%	0.71 [0.39; 1.31]
Yendewa, 2021	46	1635	61	1609	4.5%	0.73 [0.50; 1.08]
Total (95% CI)		41607		19344331	86.5%	1.06 [0.84; 1.33]
Heterogeneity: $Tau^2 = 0.1652$; $Chi^2 = 99.46$, df = 19 ($P < 0.01$); $I^2 = 81\%$						
**Africa**						
Boulle, 2020	115	3978	510	18330	4.5%	1.04 [0.85; 1.28]
Parker, 2020	6	24	22	89	4.4%	1.02 [0.36; 2.88]
Jassat, 2021	3407	34104	30697	34104	4.5%	0.01 [0.01; 0.01]
Total (95% CI)		38106		52523	13.5%	0.23 [0.00; 134.12]
Heterogeneity: $Tau^2 = 10.0993$; $Chi^2 = 1807.26$, df = 2 ($P = 0$); $I^2 = 100\%$						
**Total (95% CI)**		**79713**		**19396854**	**100.0%**	**0.81 [0.47; 1.41]**
Heterogeneity: $Tau^2 = 9.4871$; $Chi^2 = 10947.49$, df = 22 ($P = 0$); $I^2 = 100\%$						
Test for subgroup differences: $Chi^2 = 1.05$, df = 1 ($P = 0.31$)						

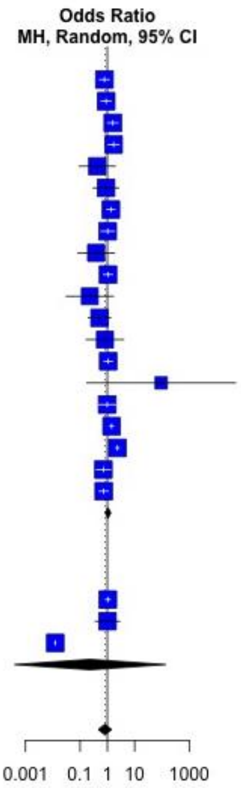

Figure 8. Mortality risk according to country location (Africa vs Outside Africa)

Publication Bias

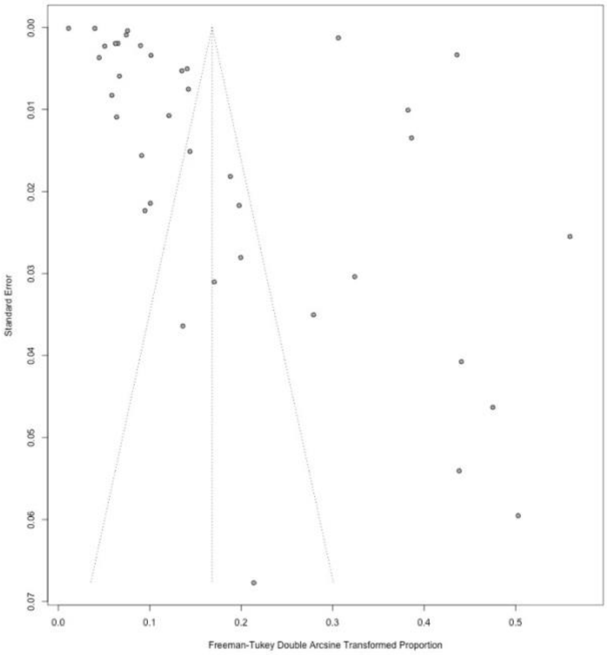

Egger's test p-value = 0.0115

Figure 9. Funnel plot of studies included in the meta-analysis of prevalence

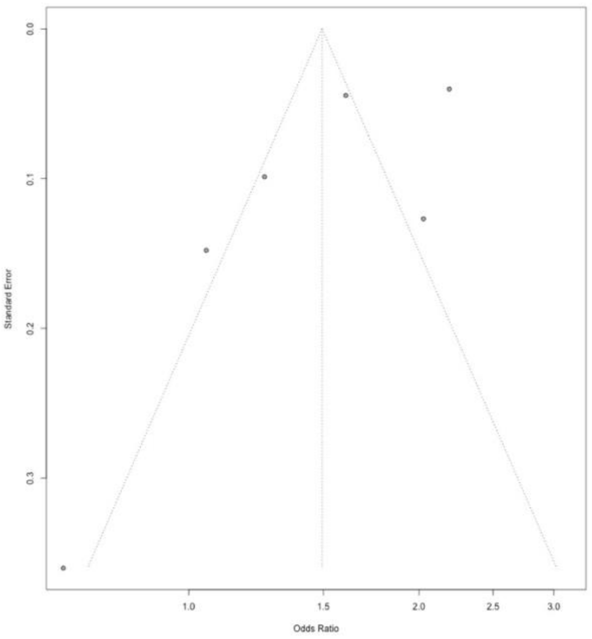

Egger's test p-value =0.1826

Figure 10. Funnel plot of studies included in the meta-analysis of In-hospital admission

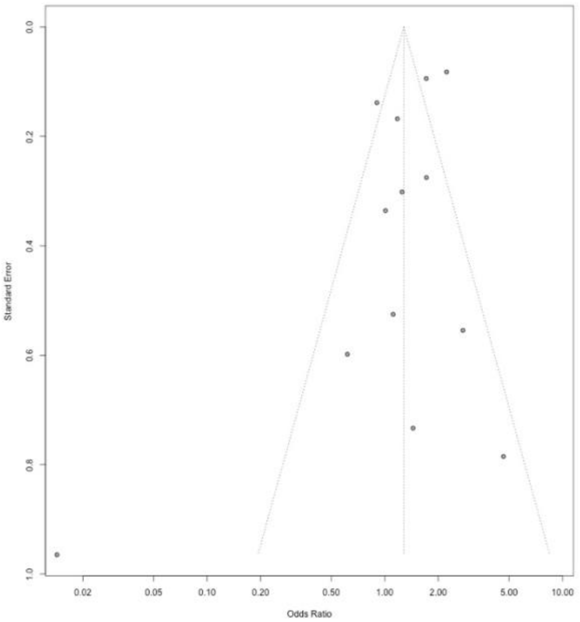

Egger's test p-value =0.1218

Figure 11. Funnel plot of studies included in the meta-analysis of severity assessment

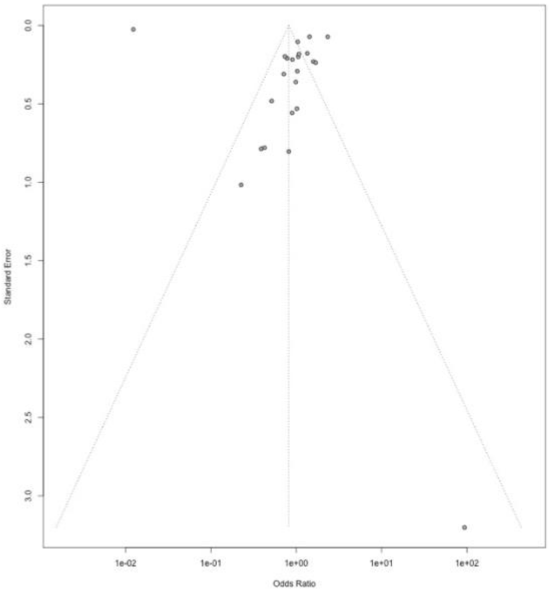

Egger test p-value = 0.0032

Figure 12. Funnel plot of studies included in the meta-analysis of mortality

Sensitivity analysis

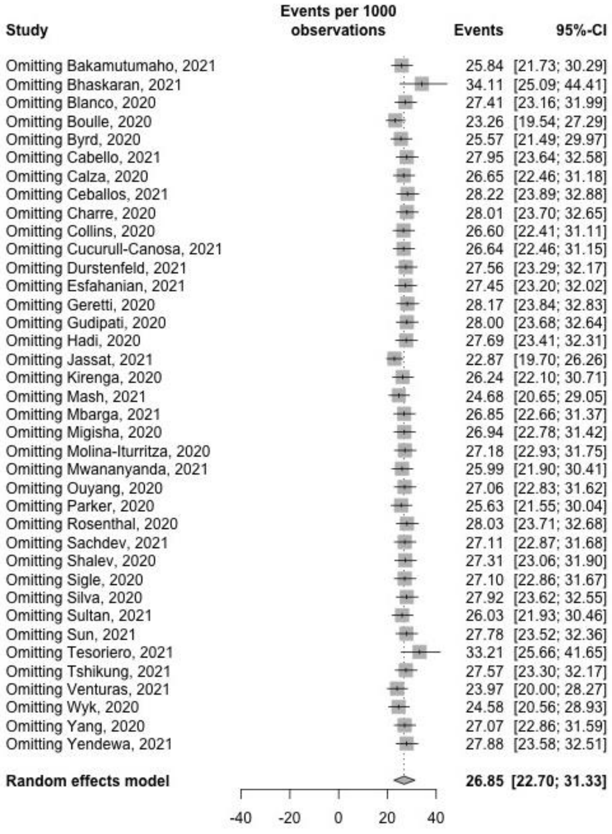

Figure 13. sensitivity analysis of studies included in prevalence assessment meta-analysis

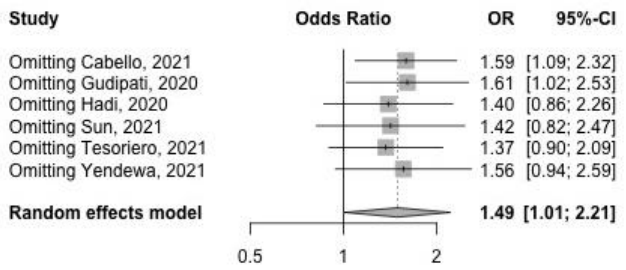

Figure 14. sensitivity analysis of studies included in in-hospital admission risk meta-analysis

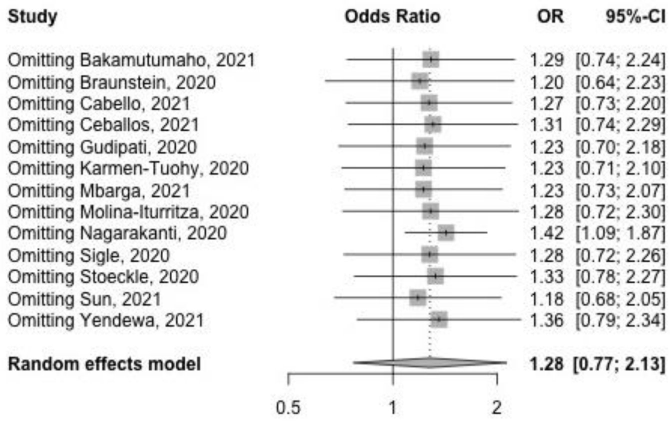

Figure 15. sensitivity analysis of studies included in Severity assessment meta-analysis

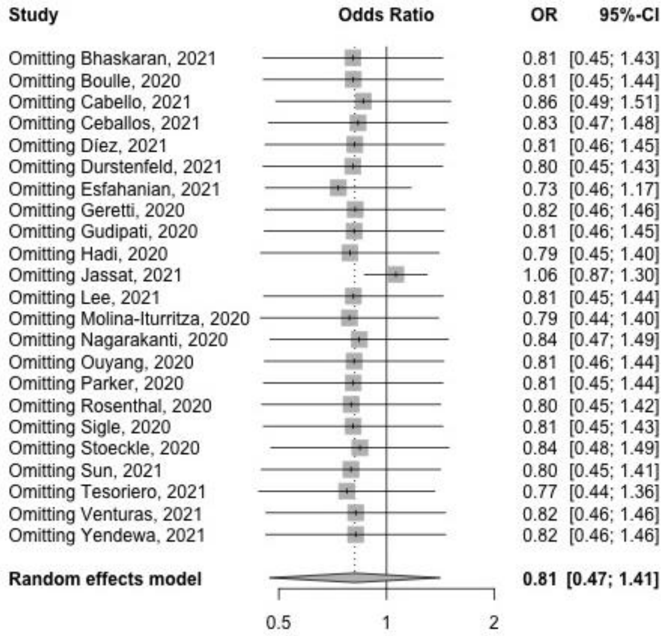

Figure 16. sensitivity analysis of studies included in mortality risk assessment
